# Avoiding Sinus Floor Elevation by Placing a Palatally Angled Implant: A Morphological Study Using Cross-Sectional Analysis Determined by CBCT

**DOI:** 10.3390/diagnostics14121242

**Published:** 2024-06-13

**Authors:** Doğan Ilgaz Kaya, Samed Şatır, Beyza Öztaş, Hasan Yıldırım

**Affiliations:** 1 Faculty of Dentistry, Oral and Maxillofacial Surgery, Karamanoglu Mehmetbey University, Karaman 70200, Turkey; 2 Faculty of Dentistry, Oral and Maxillofacial Radiology, Karamanoglu Mehmetbey University, Karaman 70200, Turkey; samedsatir@kmu.edu.tr; 3 Faculty of Dentistry, Oral and Maxillofacial Surgery, İstanbul Kent University, Istanbul 34433, Turkey; beyzadonmez@kmu.edu.tr; 4 Kamil Ozdag Faculty of Science, Department of Mathematics, Karamanoglu Mehmetbey University, Karaman 72100, Turkey; hasanyildirim@kmu.edu.tr

**Keywords:** avoiding sinus lift, palatal angled implant, virtual implant, virtual planning

## Abstract

Backgrounds: Tooth loss in the posterior maxilla often necessitates dental implant placement, but the maxillary sinus anatomy poses challenges, especially during sinus floor elevation. Mesially angled implants are an alternative for total edentulism, but for single tooth deficiencies, palatally angled implants may offer a solution. This study evaluates the prevalence of avoiding sinus floor elevation by placing palatally angled implants in cases with a single missing tooth. Methods: A retrospective study at Ahmet Keleşoğlu Faculty of Dentistry involved 100 participants with a single missing tooth and prior CBCT scans. Virtual implants were placed using OnDemand3D (version 1.0.7462) software. On CBCT sections, implants were angled palatally to avoid sinus or nasal cavity perforation. Statistical analysis was conducted using R and MedCalc (version 4.3.2) software. Results: Of the participants (60% female, average age 50.45), 76 edentulous regions required sinus elevation. The implant placeability rates varied across zones (second molar: 39.3%, first molar: 63.1%, second premolar: 78.5%). Implant placement at a palatal angle was significantly higher in the second premolar and first molar regions. Statistically significant differences were observed in the implant placeability between regions. Conclusions: This study supports the feasibility of avoiding sinus floor elevation through palatally angled implants in specific cases, reducing the associated complications.

## 1. Introduction

After tooth loss in the posterior maxilla, one of the most important limitations for dental implant application is the maxillary sinus anatomy. The sinus floor elevation performed to place dental implants in this area may cause some complications [1]. These complications may cause the placed dental implant to not be osseointegrated; it may also cause problems such as sinus infections, oroantral fistulas, or failure of the maxillary sinus to function normally [2,3]. It is recommended to use cone-beam computed tomography (CBCT) images to reduce complications of maxillary sinus floor elevation and to understand the anatomy of the region [4].

Over the last 10 years, dental implant brands have offered prosthetic solutions to implant practitioners by producing abutment and multiunit options suitable for angled implants. In cases of total edentulism, sinus floor elevation can be avoided by placing mesially angled implants [5]. Studies on this method show successful results [6]. However, in cases of single tooth deficiencies, the roots of the adjacent teeth make it impossible to apply mesially angled implants. In their case report, Lim et al. showed that they could avoid sinus floor elevation in cases where a single tooth was missing by placing the implant at a palatal angle [7]. There is no other study in the literature evaluating the possibility of placing implants using the bone in the palatal region with this method.

The aim of this study is to evaluate the frequency of avoiding sinus floor elevation surgery by placing a palatally angled implant in individuals with a single missing tooth.

## 2. Materials and Methods

This retrospective study was conducted at Ahmet Keleşoğlu Faculty of Dentistry, with approval from the Karamanoğlu Mehmetbey University Faculty of Medicine Ethics Committee (#06-2023/06). This study was conducted in accordance with the 1975 Declaration of Helsinki, as revised in 2013. In the present study, orthopantomography images (OPGs) and CBCT data from 100 participants (60 women and 40 men), whose average age was 50.45 (19–88) years, who had a single missing tooth, and who had previously had a CBCT scan, were evaluated. Patients who were older than 18 years of age with a single missing tooth in the maxilla were included. Those with pathology such as cysts or tumors, who had undergone tooth extraction within the last 3 months, who had previously undergone sinus augmentation, and who had artifacts in their tomography data were not included in the study.

**Figure 1:**
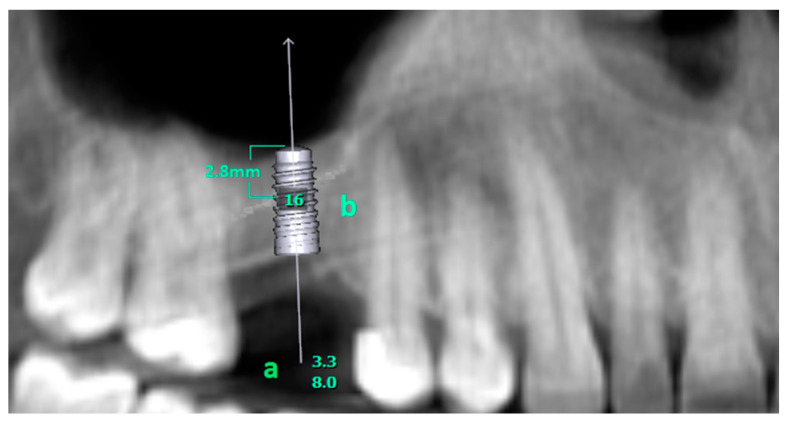
(a) shows the length and diameter of the virtually placed implant. (b) Number of the missing tooth. The virtually placed implant perforates the maxillary sinus 2.8 mm from the inferior edge.

OPGs of 578 patients who had CBCT scans were examined. One hundred patients with missing teeth in the posterior region of the maxilla were identified. Virtual implants (Straumann 3.3–8 mm, BLT, Basel, Switzerland) were placed on the OPGs of these patients using the implant planning program (OnDemand3D version 1.0.7462 software, Cypermed Inc., Seoul, Republic of Korea) (Figure 1). The angled section of the implant was 5.3 mm long and had a 9-degree angle. The platform width was 3.1 mm, and the connection point had a diameter of 2.8 mm. It also had a 0.8 mm thread pitch, a 20-degree flank lead, and a depth of 0.3 mm. This was applied to indicate sinus floor elevation. CBCT slices of participants with a residual alveolar crest height below 8 mm were examined. CBCT data of 80 edentulous regions that perforated the maxillary sinus from the inferior border and therefore required internal or external sinus elevation were examined in the sagittal plane.

**Figure 2:**
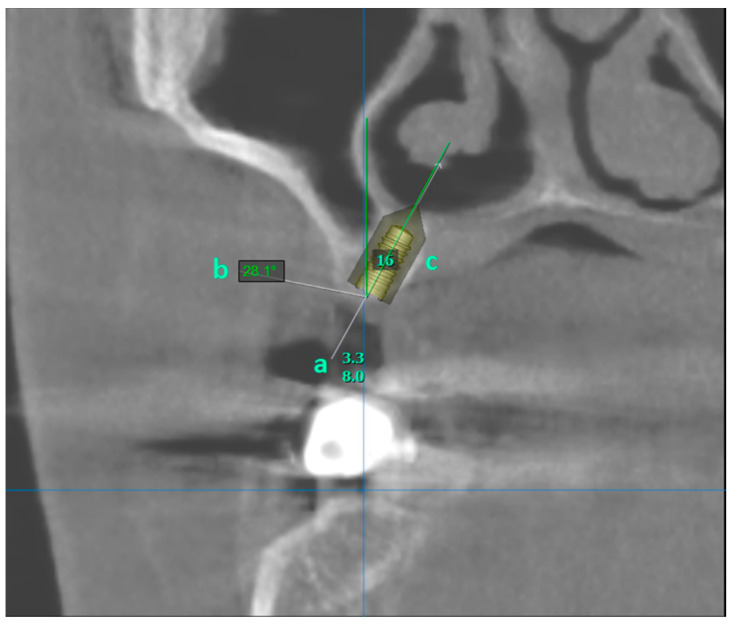
(a) shows the length and diameter of the virtually placed implant. (b) shows the angle be-tween the vertical plane and the long axis of the implant. (c) Number of the missing tooth. The virtually placed implant is completely within the bone.

Implants were placed in the edentulous area in the CBCT images. Meanwhile, it was angled in the palatal direction so as not to perforate the maxillary sinus or nasal cavity and to remain completely within the bone. Implants that perforated the maxillary sinus or the nasal cavity and implants at an angle of more than 30 degrees in the palatal direction were recorded as invalid. Implants that remained completely within the bone and at an angle of less than 30 degrees to the long axis of the adjacent tooth were recorded as valid (Figure 2).

To ensure accuracy in the measurements, the scale bar and the implant template of the CBCT images were calibrated. Two researchers (B.Ö., D.K.) were asked to determine the optimal position for the positioning of the virtual implants. In cases of disagreement, it was re-evaluated and discussed, and a consensus was reached by taking the opinion of a third researcher (S.Ş.).

### Statistical Analysis

The statistical analysis stage of the study covers descriptive statistics and significance comparisons for means and ratios. The quantitative measurements were presented as means and standard deviations, while qualitative data were presented as frequency and percentage values. The mean comparisons were assessed via the independent samples *t*-test, with the relationships between categorical measures evaluated via the chi-square test and the ratio comparisons via the binomial test based on the z-test. The comparison results of the quantitative measurements are additionally visualized with a box–whiskers plot. All the statistical analyses were performed with R (version 4.3.2) and MedCalc (version 23) software. In the study, the significance level was taken constant as 0.05.

## 3. Results

Due to the scope of the study, it is mainly descriptive, yet it is supported by a number of inferential results, including group comparisons and independence tests. The results on the descriptive statistics of the measurements used in the study are presented in Table 1 and Table 2. According to the results, 60% of the participants were female, and the overall average age was 50.45 ± 13.97 years. There was no significant difference between the mean ages in different gender groups (*p* = 0.357).

The counts and ratios of participants diagnosed as compatible for implantation with the measurements from different regions are shown in Table 3. An overall comparison of the homogeneity of the spread of the implant placeability in different zones was evaluated by using the chi-square homogeneity test, and the results are shown in Table 4. The rates on the implant placeability for each region are compared pairwise with the binomial test based on the z-test and given in Table 5. According to the results in Table 4 and Table 5, there are statistically significant results in the ratios between region and implant placeability in general (*p* = 0.033), which are also found as statistically significant differences on the basis of region (*p* < 0.001). The percentages of compliant and non-compliant patients were significantly different in the three zones (*p* < 0.001).

## 4. Discussion

Nowadays, mesially angled dental implant placement has become part of the clinical routine in patients who cannot undergo bone augmentation due to various systemic problems [8]. In addition, implants placed at an angle to the mesial also have some biomechanical advantages [9]. However, in single tooth deficiencies, the mesial tooth makes it impossible to have an angled implant. At this point, placing the implant at a palatal angle may provide an advantage to the clinician. In the present study, it was investigated whether it is possible to avoid this procedure by placing palatally angled implants in patients with a sinus lift indication.

Sinus floor elevation is a frequently used method in implant surgery. It has been proven in various systematic reviews that implants placed after this procedure have a survival rate of more than 90% [10,11,12,13,14,15]. However, it is also known that direct and indirect sinus floor elevation causes some complications, such as membrane perforation, chronic rhinosinusitis, hemorrhage, ostium obstruction, benign paroxysmal positional vertigo, and implant displacement [1]. However, sinus floor elevation is a challenging process in terms of cost and procedural difficulty. The present study has shown that it is possible to avoid sinus floor elevation in some patients.

In the current study, it was observed that the rate of implant placement at a palatal angle was statistically higher in the second premolar and first molar teeth regions than in the second molar region. In their study, Lim et al. found that after the extraction of the second premolar, first molar, and second molar teeth, the greatest sinus pneumatization was in the second molar tooth region [16]. These data are compatible with the present study. Less sinus pneumatization allows sufficient bone to remain in the alveolar crest for implant placement in the palatal region. The direction of sinus pneumatization may also hinder implant placement. In their study, Yücesoy et al. showed that just like pneumatization in the inferior direction, pneumatization in the medial direction would require an internal or external sinus elevation operation [17]. In this study, care was taken to ensure that the apical region of the implants remained completely within the alveolar bone. Cases with anatomical features showing pneumatization in the medial direction were considered invalid.

In the case report of Lim et al., which inspired the present study, edentulousness was rehabilitated by placing implants at a palatal angle in tooth areas 15 and 16 [7]. In this case, it appears that a surgical guide was used for the implant placement. The issues of pain reduction, surgery time, and cost in the use of surgical guides are currently controversial [18]. However, since it is difficult to place implants at a palatal angle in free-hand surgeries, a surgical guide is needed.

In angled abutments, it has been observed that abutments with an angle of less than 30 degrees give better results in terms of the bone loss around the implant [19]. For this reason, in the present study, we determined that implants placed at an angle of more than 30 degrees in the vertical plane were invalid. In a finite element study conducted by Gümrükçü et al., it was predicted that less marginal bone loss would be seen in 45-degree-angled implants compared to 30-degree-angled implants due to the decrease in the cantilever length [20]. If this situation is proven by other studies in the future, it may be possible for implants placed at an angle in the palatal direction to be applied in more cases. For angled implants, cement-type prosthetic rehabilitation options are also available, in addition to screw-supported restorations. Studies have shown that resin-based cements have high mechanical properties. Thanks to advances in the cementation materials, prosthetic restorations of angled implants will also have high mechanical properties in the future [21].

The most important limitation of this study is that the relationship of the restorations to the lower jaw was not evaluated while placing the implants in the virtual environment. The angle of the abutment to be used may need to be higher to ensure ideal occlusion. This may prevent the placement of a palatally angled implant. In addition, only one model of a single brand was placed virtually in the study. Implants of different brands with different dimensional properties could also have been evaluated in the study.

## 5. Conclusions

In light of the data obtained from this study, in some cases where direct or indirect sinus elevation is anticipated, this procedure may be omitted by using implants placed at an angle to the palate. According to the results of the study, it is statistically possible to place implants at an angle to the palate, especially in the second premolar and first molar tooth regions. In this way, some complications that may develop due to sinus elevation surgery can be prevented. For this reason, it would be useful to re-evaluate the CBCT data of patients for whom sinus elevation is indicated.

## Figures and Tables

**Table 1:** The descriptive statistics of demographic variables.

		**Mean**	**Standard Deviation**
Age		50.45	13.97
		***n***	**Percentage**
Gender	Male	40	40%
	Female	60	60%

**Table 2:** The comparison of age measurements across gender.

		Age			
		Mean	Standard Deviation	t	*p*
Gender	Male	48.88	13.63	−0.926	0.357
	Female	51.53	14.20		

**Table 3:** The implant placeability rates for different zones.

Region	Measurement	Count	Percentage	Available		Not Available	
				*n*	Percentage	*n*	Percentage
Second Molar	Edentulous	28	14%	11	39.3%	17	60.7%
	Dentulous	172	86%				
First Molar	Edentulous	38	19%	24	63.1%	14	26.9%
	Dentulous	162	81%				
Second Premolar	Edentulous	14	7%	11	78.5%	3	21.5%
	Dentulous	186	93%				

**Table 4:** The statistical comparison results on the homogeneity of the region and implant placeability condition.

Region	Available		Not Available		X^2^	*p*
	*n*	Percentage	*n*	Percentage		
Second Molar	11	39.3%	17	60.7%	6.843	0.033
First Molar	24	63.1%	14	26.9%		
Second Premolar	11	78.5%	3	21.5%		

**Table 5:** The statistical comparison results on the ratio of implant suitability in each region.

Region	Available		Not Available		z	*p*
	*n*	Percentage	*n*	Percentage		
Second Molar	11	39.3%	17	60.7%	−2.83	0.005
First Molar	24	63.1%	14	26.9%	3.68	<0.001
Second Premolar	11	78.5%	3	21.5%	7.92	<0.001

## Data Availability

The datasets used and/or analyzed during the current study are available from the corresponding author on reasonable request.
